# Prebiotic Syntheses of Organophosphorus Compounds from Reduced Source of Phosphorus in Non-Aqueous Solvents

**DOI:** 10.3390/life13112134

**Published:** 2023-10-29

**Authors:** Maheen Gull, Tian Feng, Benjamin Smith, Laurent Calcul, Matthew A. Pasek

**Affiliations:** 1 School of Geosciences, University of South Florida, 4202 E. Fowler Ave. NES 204, Tampa, FL 33584, USA; tianfeng1@usf.edu (T.F.); mpasek@usf.edu (M.A.P.); 2 Department of Chemistry, University of South Florida, 4202 E. Fowler Ave. CHE 205, Tampa, FL 33620, USA; calcul@usf.edu (L.C.); benjamins216@usf.edu (B.S.)

**Keywords:** organophosphorus compounds, origin of life, non-aqueous solvents, condensation, phosphorylation, biological esters, phosphite

## Abstract

Reduced-oxidation-state phosphorus (reduced P, hereafter) compounds were likely available on the early Earth via meteorites or through various geologic processes. Due to their reactivity and high solubility, these compounds could have played a significant role in the origin of various organophosphorus compounds of biochemical significance. In the present work, we study the reactions between reduced P compounds and their oxidation products, with the three nucleosides (uridine, adenosine, and cytidine), with organic alcohols (glycerol and ethanolamine), and with the tertiary ammonium organic compound, choline chloride. These reactions were studied in the non-aqueous solvent formamide and in a semi-aqueous solvent comprised of urea: ammonium formate: water (UAFW, hereafter) at temperatures of 55–68 °C. The inorganic P compounds generated through Fenton chemistry readily dissolve in the non-aqueous and semi-aqueous solvents and react with organics to form organophosphites and organophosphates, including those which are identified as phosphate diesters. This dual approach (1) use of non-aqueous and semi-aqueous solvents and (2) use of a reactive inorganic P source to promote phosphorylation and phosphonylation reactions of organics readily promoted anhydrous chemistry and condensation reactions, without requiring any additive, catalyst, or other promoting agent under mild heating conditions. We also present a comparative study of the release of P from various prebiotically relevant phosphate minerals and phosphite salts (e.g., vivianite, apatite, and phosphites of iron and calcium) into formamide and UAFW. These results have direct implications for the origin of biological P compounds from non-aqueous solvents of prebiotic provenance.

## 1. Introduction

Phosphorus (P) plays a significant role in all living forms as an essential component that is involved in metabolism and biochemical reactions [1,2]. Ionized phosphate esters are ubiquitous in biochemistry for two reasons; (1) metabolites should be charged to prevent the loss of these compounds from the lipid-based cell-membrane, and (2) the charge must be negative so as to repel nucleophiles, therefore being able to resist breakdown by hydrolysis [1,2]. Phosphate uniquely allows for these requirements [3,4,5,6]. Phosphorus is hence considered to have played a key role in the origin of life on the early Earth as suggested previously [7,8,9,10]. On the early Earth, P is assumed to have been present mainly in the form of phosphates (including orthophosphate minerals) such as apatite (Ca_5_(PO_4_)_3_(F,Cl,OH)), whitlockite (Ca_9_(Mg,Fe)(PO_4_)_6_PO_3_OH), and brushite (CaHPO_4_·2H_2_O) [4,9,11]. 

Calcium phosphate minerals are the dominant form of phosphates [12]. These phosphate minerals that are considered to be prebiotically relevant [4,12] are poorly soluble in water and as such react poorly with organics. The liberation of P (as phosphate) from rocks takes place by the dissolution of various phosphate minerals such as apatite. Mineral dissolution is pH-dependent [12] and under typical pH (~7), P is minimally available. This low solubility and reactivity of the phosphate minerals [13,14,15] is considered to be an issue in the realm of prebiotic chemistry, known as, “the phosphate problem” [16]. This problem could plausibly have directly impacted the event of prebiotic phosphorylation on the early Earth, as the C-O-P linkage formation requires condensation reactions that are thermodynamically disfavored [17], considering water as the major solvent on the early Earth. Therefore, the prebiotic formation of the P compounds of biological relevance has been challenging [18]. 

One prebiotically plausible alternative route to address the low reactivity of P towards various organics is the use of non-aqueous solvents in lieu of water. If water is removed by evaporation from a warm pond containing prebiotic reagents, phosphorylation can readily occur [12]. Non-aqueous solvents similarly promote condensation, leading to prebiotic phosphorylation. Formamide (HCONH_2_) has been suggested to be one of the earliest, prebiotically relevant anhydrous solvents [15]. This organic compound is both a reactant and a solvent under prebiotic conditions [19,20,21,22]. The route to the prebiotic formation of formamide has been suggested by the use of simple precursor molecules such as HCN, NH_3_, and CO [23,24]. Moreoever, it has also been detected in the interstellar medium [25]. Another example of prebiotically relevant anhydrous solvents includes deep eutectic solvents such as mixtures of urea and choline chloride [26,27,28,29]. Possibly, to date, one of the most prebiotically plausible solvents is proposed to be a mixture of urea, ammonium formate, and water [30]. The work by Burcar and colleagues showed efficient phosphorylation of nucleosides in this solution mixture, even when apatite was used as a phosphorylation agent [30]. Heating this semi-aqueous solvent mixture (urea, ammonium formate, and water) at 70 °C is known to partially transform the ammonium formate to formamide, thus indicating promise for anhydrous conditions required for phosphorylation [30].

Another route to the facile formation of organophosphorus compounds is the use of reduced-oxidation-state P compounds (reduced P, hereafter) [16,31]. These inorganic reduced P compounds can be about 10^3^–10^6^ times more soluble in water compared to orthophosphate in the presence of divalent cations [3]. The prebiotic plausibility of reduced P compounds on the early Earth is supported by the detection of phosphonic acids in the Murchison meteorite [32], phosphite in the hydrothermal environments [33], interstellar synthesis of phosphorus oxoacids [34], the natural reduction of phosphate into phosphite [35], and prebiotic syntheses of several phosphonic acids [36]. An additional source of these reduced P compounds is extraterrestrial impacts that delivered the meteoritic mineral screibersite (Fe,Ni)_3_P [37], which releases various inorganic P species upon aqueous corrosion and is considered to be a significant source of various organophosphorus compounds of prebiotic origin [38]. 

These reduced P compounds also undergo a condensation reaction in the presence of urea and under mild heating conditions (i.e., heating to dryness), and form energetic condensed reduced P compounds, including pyrophosphite and the mixed-valence compounds isohypophosphate [39]. These high-energy condensed P compounds react with organics to form organophosphorus compounds [39].

The reduced P compounds oxidize into phosphate PO_4_^3−^ (1) in the presence of ultraviolet light and H_2_S/HS^−^, via a thiophosphate intermediate [40], (2) by auto-oxidation under mild heating and in the presence of condensation agents [39], and (3) by oxidation with H_2_O_2_ catalyzed by Fe^2+^ [41], called the Fenton reaction. This Fenton reaction produces reactive ·OH and ·OOH radicals that oxidize reduced P compounds by cleaving the H-P bond to generate a phosphite radical. Phosphite radicals are disproportionated to phosphate (PO_4_^3−^) and condensed phosphates such as pyrophosphate (HP_2_O_7_)^3−^, triphosphate (H_3_P_3_O_10_)^2−^, and trimetaphosphate (P_3_O_9_^3−^) [41]. 

The Fenton reaction requires H_2_O_2_, which would have been a strong oxidant in the anoxic prebiotic environments [42]. Peroxide could have formed via photolysis of atmospheric water [43,44] or ice [42]. Dry, cold, and low-oxygen conditions would have promoted the formation of H_2_O_2_ through photolysis reactions of H_2_O in Archean atmospheres [42,45,46]. Water ice that would have been part of glaciers during “Snowball Earth” events [42] could also have undergone photolysis to form H_2_O_2_. Another route to forming H_2_O_2_ involves the abrasion of quartz surfaces, which would form reactive free radicals that could oxidize water to H_2_O_2_ and O_2_ [47].

In our previous studies, we demonstrated that inorganic P compounds generated through Fenton chemistry of hypophosphite actively react with nucleosides in water in the presence of urea and NH_4_^+^ to generate phosphite and phosphate esters, including dimers (nucleoside-phosphate-nucleoside) and cyclic organic phosphates [48]. In the present study, we report plausible Fenton reactions of hypophosphite in non-aqueous solvents such as formamide and a semi-aqueous solvent composed of urea, ammonium formate, and water (UAFW, hereafter). We show that reduced P compounds and their oxidation (P) products generated via Fenton reactions can react with organics in non-aqueous and semi-aqueous solvents to form organophosphorus compounds of biological significance. We also investigate the release of inorganic P from various prebiotically relevant phosphite minerals, i.e., phosphites of calcium and iron, into non-aqueous solvents. Finally, we also compare the release of P in these iron and calcium phosphite minerals with their phosphate counterparts such as vivianite and apatite under the same conditions, and also compare the molarities of the respective solutions. 

## 2. Materials and Methods

Chemical reagents for the reactions included: sodium hypophosphite hydrate (NaH_2_PO_2_·H_2_O, 98%), ethanolamine (C_2_H_7_NO, ≥ 98%), choline chloride ((CH_3_)_3_N(Cl)CH_2_CH_2_OH, ≥ 98%), and formamide (99%). Standard compounds include uridine-5-monophosphate (5′-UMP), adenosine-5-monophosphate (5′-AMP), 2′,3′-cyclic AMP and 3′,5′-cyclic AMP, cytidine-5-monophosphate (5′-CMP), glycerol phosphate disodium salt hydrate (isomeric mixture), and phosphoethanolamine, and were purchased from Sigma Aldrich. Sodium hydroxide (NaOH, 98.5%), phosphorous acid (H_3_PO_3_, 98%), adenosine (C_10_H_13_N_5_O_4_, 98%), uridine (C_9_H_12_N_2_O_6_, 98%), cytidine (C_9_H_13_N_3_O_5_, 98%), glycerol (C_3_H_8_O_3_, 99%), and deuterium oxide (D_2_O, 99.8% atom %D) were from Acros Organic. Other reagents such as ammonium hydroxide (NH_4_OH, 25% solution in water), calcium chloride (CaCl_2_, 98%), and ferrous chloride tetrahydrate (FeCl_2_·4H_2_O, 98%) were purchased from Alfa Aesar. Hydrogen peroxide (H_2_O_2_, 30% *v*/*v*) was from Fisher Scientific. Vivianite (Fe_3_(PO_4_)_2_·8H_2_O), and apatite (Ca_5_(PO_4_)_3_(F, Cl, OH)) were purchased from Amazon.

Deionized water (DI, hereafter) was obtained in-house by using a Barnstead (Dubuque, IA, USA) NANO pure^®^ Diamond Analytical combined reverse osmosis-deionization system [39]. The semi-aqueous solvent UAFW was prepared as in prior studies by using a 1:2:4 molar ratio of urea: ammonium formate: water [30]. This mixture was transferred to a glass vial of 20 mL capacity and was sealed, followed by heating at 65 °C until dissolved. The consistency of the UAFW solvent was transparent and all the contents were completely dissolved. The solvent was prepared and was stored at 4 °C. The initial pH of the solvent was found to be ~7.5 using pH paper purchased from Hydrion paper. After heating, the pH of the eutectic was measured at pH 5.5–6.0, which remained consistent over the course of the experiments. The other non-aqueous solvent, i.e., formamide, was used as purchased. 

Iron phosphite (FeHPO_3_) was synthesized as previously [48]. Equimolar solutions of FeCl_2_·4H_2_O and H_3_PO_3_ (0.1 M each) were mixed slowly, and the mixture was stirred with the help of a magnetic stirrer. On mixing, brownish precipitates were formed and immediately filtered, dried, and stored for future use. Calcium phosphite (CaHPO_3_) was prepared by mixing equimolar solutions of CaCl_2_·4H_2_O and Na_2_HPO_3_.5H_2_O (0.1 M each). A white precipitate was separated through filtering the solution, dried, and stored for future use. The other two minerals, i.e., vivianite and apatite, were crushed into fine powders and were stored in vials for future use.

### 2.1. Oxidation of Hypophosphite by Fenton Reaction

**Figure 1:**
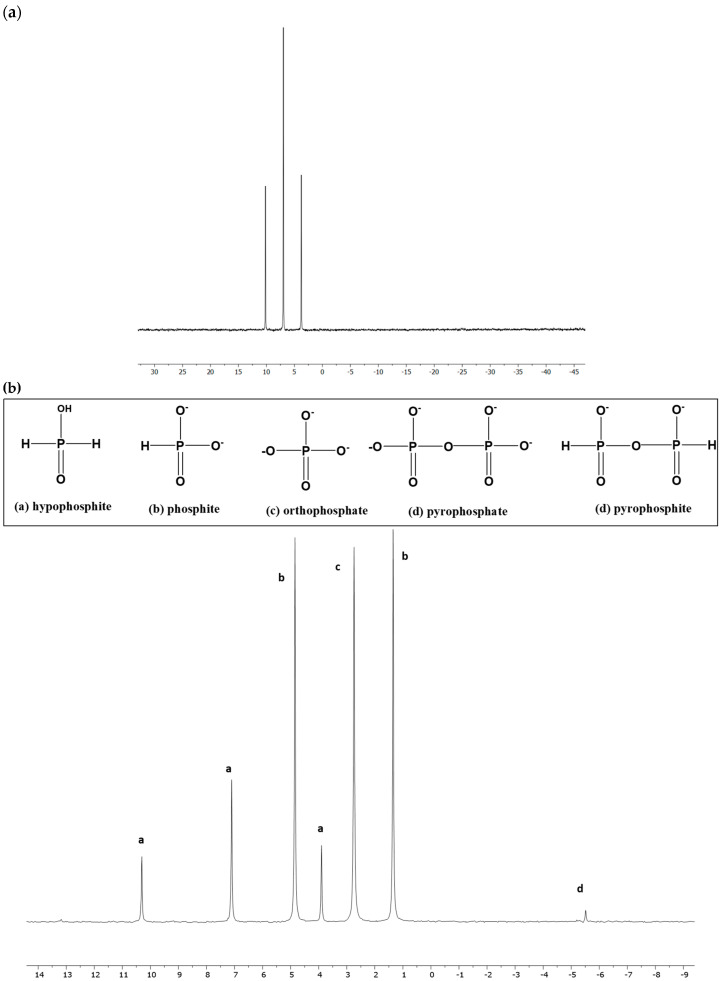
(**a**) H-coupled, ^31^P-NMR spectrum of starting material (sodium hypophosphite) showing a wide triplet. (**b**) H-coupled ^31^P-NMR spectrum of the inorganic P compounds after the Fenton reaction. The starting reduced P source is hypophosphite (**a**). The y-axis represents the signal strength (%) while x-axis represents δ (ppm).

The pH of the starting DI (deionized) water was around 6. Sodium hypophosphite (NaH_2_PO_2_) was used as a source of the reduced P material for the Fenton reaction to generate various oxidized forms of inorganic P, along with the condensed phosphates. ^31^P-NMR analysis of the starting material showed no other P peaks as impurities (Figure 1a). Fenton reactions were formed by the previously reported method [41,48]: aqueous solution of equimolar (0.2 M of both) hypophosphite (H_2_PO_2_^−^) and FeCl_2_.4H_2_O (equal volumes, 0.1 M total of each reagent) were mixed and dissolved to form an homogenous solution. The total volume of this solution was 20 mL (10 mL for each of the solutions described above). To this solution mixture, 15 mL of 0.50 M H_2_O_2_ was added dropwise.

In our study, the concentration of H_2_O_2_ was varied from 0.1–0.5 M to study the extent of the formation of the oxidized P compounds generated by the Fenton reactor [41]. This mixture was sealed and was allowed to stir at room temperature by using a magnetic stirrer for 24 h. After this time, the initial pH of the solution was found to be around 4.0–4.5. This mixture was subsequently quenched and titrated by 20% ammonium hydroxide (NH_4_OH), and was sealed immediately to prevent the escape of NH_3_ from the NH_4_OH solution, followed by stirring on a magnetic stirrer at room temperature. The final volume of the solution after adding NH_4_OH solution was about 45–50 mL with a pH = 11–12.5. Thick orange-brown precipitates were observed. This step indicated the separation of insoluble Fe^3+^ precipitating as Fe(III)(O,OH)_X_ compounds. This step was necessary for the separation of Fe^2+^ from Fe^3+^ for the analysis of sample by ^31^P-NMR. The resulting solution mixture was filtered with the help of a Whatman filter paper no. 1 and stored for further use as previously [48]. This filtrate was labeled as inorganic P Fenton solution (IPF solution hereafter). 

### 2.2. Syntheses of Biological P Esters by P Products from Fenton Solution 

The biomolecule substrates included nucleosides (adenosine, uridine, and cytidine), organic alcohols (glycerol and ethanolamine), and the organic quaternary ammonium compound choline chloride. These compounds and their phosphorylated derivatives are significant in biochemistry and actively take part in various metabolic pathways, such as the formation of genetic makeup, cell-membrane structure, and respiration. 

The prebiotic phosphorylation and phosphonylation reactions of organic compounds with Fenton solution were carried out by adding 4 mL of formamide or UAFW in a clean and unsealed glass vial. To this vial, 0.40–0.65 g of organic compound was also added (Table 1). Finally, 7 mL of IPF solution was also added. The pH of the solution was around 10–11. The unsealed reaction vial was then allowed to heat at 55–68 °C from 20 h to 5 days, on a hot plate with a magnetic stirrer. The reaction mixture was kept unsealed to promote evaporation of water introduced from the IPF solution to mimic a hot drying and concentrated pool on the early Earth [22,30,48,49,50]. Two reaction sets for each organic reagent, including the nucleosides, organic alcohols, and the ammonium compound, were allowed to heat (unsealed) at 55–68 °C from 20 h to 5 days, with the only difference being the type of solvent. For Set-1 of reactions, formamide was used as a solvent, while for Set-2, UAFW was used as a solvent under similar conditions.

After the completion of the reaction, the reaction sample volume was reduced to almost half from evaporation of water from the reaction mixture. The sample, however, was still in solution because of the presence of the non-aqueous or semi-aqueous solvents. 

After the required heating time, the reaction mixture was removed from heating and was allowed to cool down slowly to room temperature. It was then mixed with 5 mL DI water and stirred with a magnetic stirrer until a clear solution was formed, which was subsequently filtered through filter paper. The filtrate was transferred to a clean watch glass followed by air-drying at room temperature. This sample was allowed to concentrate overnight under ambient conditions with about ~2 mL remaining. 2 mL D_2_O (75%) and DI water (25%) were added to this solution mixture. In case of MS analyses, only DI water was used as previously described [48]. The total volume of the solution was 5 mL. About 430–350 µL of the sample solution was transferred to a clean NMR tube and analyzed via ^31^P-NMR.

A few reactions were also carried out with the ‘unquenched Fenton solution’. In such experiments, the organic was directly heated with the Fenton solution without quenching with a base (NH_4_OH or NaOH) or without bringing the pH from 4.5 to 11. Once the Fenton solution was generated (Section 2.1), it was heated with the organic substrate (7 mL and pH = 4.5) to dryness at 55–68 °C for 20 h to 5 days. Both solvents were attempted. After the completion of the reaction, the dried mixture was treated with 0.1M NaOH solution to completely precipitate out Fe^3+^ so it could be studied through ^31^P-NMR. The mixture then followed the same protocol of solution preparation and was analyzed via ^31^P-NMR.

Some blank reaction sets were also carried out, in which the IPF solution along with the solvent (formamide or UAFW) was heated (unsealed) at the same temperature as that which was used for organics phosphorylation and phosphonylation reactions (55–68 °C). No organic substrate was added in such samples. After completion, a similar quenching protocol was followed, and samples were characterized by ^31^P-NMR.

### 2.3. Studies on the Release of Inorganic P from Various Prebiotically Relevant P Minerals

The phosphate minerals selected in the studies included; vivianite (Fe_3_(PO_4_)_2_·8H_2_O) and apatite (Ca_5_(PO_4_)_3_(F, Cl, OH)) and their phosphite counterparts, iron (II) phosphite (FeHPO_3_) and calcium phosphite (CaHPO_3_). Each material was ground and crushed into a fine powder. In each case, 0.2 g of the material was taken in a clean glass, and to this vial, 4 mL of the solvent was added. The vial was capped (sealed) and was stirred by a magnetic stirrer on a hot plate at 65–68 °C (Table 2). The samples were capped to avoid the evaporation of the water from UAFW, unlike the studies in Section 2.2; the purpose of sealing this set of reactions was to study the release of P from the materials into the solvent, and not to promote condensation/evaporation reactions. The reaction vials were stirred and heated for 3 days. After 3 days, the samples were analyzed via ^31^P-NMR to study the comparative release of P into the solvents. 

### 2.4. Analyses, Identification and Characterization of Inorganic and Organic P Compounds

The samples were analyzed via ^31^P-NMR and mass spectrometry (MS). For ^31^P-NMR analyses, the samples were analyzed on a 600-MHz Bruker Neo NMR operating at 242.9 MHz in both H-coupled and H-decoupled modes. The width of the spectrum was 200 ppm, and the running temperature was 22 °C. The P products of the reactions were quantified by peak integration method as previously reported [39,48,49,50,51]. The relaxation time (D1) used between NMR scans throughout this study was set to 2 s. This was compared to several experiments run at D1 = 30 s. Since the integral values of D1 = 2 s and D1 = 30 experiments were comparable, a D1 = 2 s is considered quantitative, and the remaining experiments were run at D1 = 2 s. The sample preparation for the NMR analysis has been discussed in detail in Section 2.2.

^31^P-NMR studies in case of the reaction samples containing any insoluble mineral such as vivianite and apatite were performed as follows: After 3 days, the sealed reaction sets (Section 2.3) were removed from heating and were allowed to cool down. To each sample solution, 1 mL of DI water was added, making final volume up to 5 mL. Each sample was then centrifuged to remove the insoluble mineral. The clear contents from the solution were taken into an Eppendorf tube and D_2_O was added (50:50) *v*/*v*. Each sample was then analyzed from 450 to 1000 scans. 

The molarity [M] of the solutions for reaction Set-1 and Set-2 (Section 2.3) was calculated using the formula (Equation (1)) as suggested previously [37,51].

(1) $$[M]=0.0075\;\times \;{\left(\frac{\left(\frac{S}{N}\;\right)}{\sqrt{Scans}}\right)}^{2}+0.0007\;\times \;\left(\frac{\left(\frac{S}{N}\;\right)}{\sqrt{Scans}}\right)+0.0001\;$$

where S/N is the signal-to-noise ratio, and Scans means the number of NMR scans taken [37,51]. This relationship was empirically determined and is accurate to about 10% over the range of 10^−4^ to 10^−2^ M based on the various sample spectra obtained [37,51]. 

MS analyses were formed in negative ion mode on a 6130 Single Quadrupole Mass Spectrometer (Agilent, Santa Clara, CA, USA) attached to an Agilent 1200 HPLC by direct injection, and deionized water was used as a solvent as reported previously [48,49,50].

Some of the reaction samples were also quantified by using phosphonoacetic acid (PAA, hereafter) as an internal standard (SI, and also see ref. [48]).

Organophosphorus compounds such as 5′-AMP, 5′-UMP, 5′-CMP, glycerol phosphates, and phosphoethanolamine were confirmed by spiking with standards [48,49]. The other organophosphorus compounds, including organic phosphites, were identified and characterized by studying their characteristic peak splitting in the H-coupled ^31^P-NMR, measuring their J-coupling constants, and by finding the target peaks in the mass spectrometer as reported previously [39,48,49,50].

## 3. Results

The Fenton reaction of hypophosphite generated various oxidized (inorganic) P products, including condensed P compounds such as pyrophosphate. Figure 1 shows the H-coupled ^31^P-NMR spectrum of a Fenton solution after the completion of the reaction, followed by quenching with a base (IPF solution). Peak (a) represents a wide triplet for hypophosphite identified by comparing the coupling constant values (J); phosphite splits into a wide doublet (peak b) and was also confirmed by its coupling constant value to be around 550–570 Hz [48,51], orthophosphate as a singlet peak (peak c), and pyrophosphate as a singlet around −5 to −6 ppm. This IPF solution readily reacted with organics, and on heating with organic substrates (in non-aqueous solvents) at 55–68 °C resulted in the formation of various organophosphorus species. Condensed P compounds such as pyrophosphate were observed when the quenched Fenton solution (IPF solution) was mixed with formamide or UAFW and was heated at 68 °C for 3–4 days (without organics) (Table 1, first two entries). Various organophosphorus compounds were observed. For nucleosides (uridine, adenosine, and cytidine), alcohols (glycerol and ethanolamine), and choline chloride, both phosphate and phosphite derivatives were observed Supplementary Materials. 

Organophosphorus products were identified by peak splitting, peak location (ppm), and, when available, spiking with authentic standards. Organophosphorus compounds were also confirmed by MS (the direct injection method) as in our previous studies [29,48,49,50]. The MS analyses of reaction samples containing glycerol and IPF showed the following major peaks: [C_3_H_9_O_5_P-H] at *m*/*z* 155.02 corresponding to glycerol phosphite, and [C_3_H_10_O_7_P_2_-H] at *m*/*z*: 218.99 corresponding to glycerol diphosphite (two different phosphite groups attached at different location on the glycerol molecules, rather than a pyrophosphite (P-O-P) linkage) [39].

In the reaction samples with uridine with IPF solution, the following peaks were observed: [C_9_N_2_O_6_H_11_-H] at *m*/*z* 243 corresponding to unreacted uridine nucleoside, [C_9_N_2_O_9_PH_13_-H] at *m*/*z* 323.04 corresponding to uridine-monophosphate (2′, 3′ and 5′-UMP compounds), [C_9_H_11_N_2_O_8_P-H] at *m*/*z* 305 corresponding to 2′,3′-cyclic UMP, and [C_9_N_2_O_8_PH_12_-H] at *m*/*z* 307 corresponding to uridine-monophosphite [48]. Also, the major peaks in MS for the adenosine reaction with IPF solution in the non-aqueous solvents were as follows: [C_10_H_13_N_5_O_4_-H] at *m*/*z* 266 corresponding to unreacted adenosine nucleoside, [C_10_H_13_N_5_O_7_P-H] at *m*/*z* 346 corresponding to monophosphate (2′, 3′ and 5′-AMP compounds), [C_10_H_11_N_5_O_6_P-H] at *m*/*z* 327 corresponding to adenosine 2′,3′-cyclic monophosphate, and [C_10_H_14_N_5_O_6_P-H] at *m*/*z* 330 corresponding to adenosine-monophosphite. These reactions were also compared with our previous studies on these compounds [39,48]. 

Similarly, the reaction samples containing cytidine (with IPF) were also studied, as were peaks corresponding to [C_9_H_13_N_3_O_5_-H] at *m*/*z* 242 (unreacted cytidine nucleoside), [C_9_H_14_N_3_O_8_P-H] at *m*/*z* 322 corresponding to cytidine monophosphate (2′, 3′ and 5′-CMP) compounds, [C_9_H_14_N_3_O_7_P-H] at *m*/*z* 306 corresponding to cytidine monophosphite species (including 2′, 3′ and 5′- species), and [C_9_H_12_N_3_O_7_P-H] at *m*/*z* 304 corresponding to cytidine-2′,3′-cyclic monophosphate.

In the reaction samples containing IPF and choline chloride in the non-aqueous solvents, the major peaks identified were as follows: [C_5_H_14_NO_4_P-H] at *m*/*z* 183 corresponding to phosphocholine and [C_5_H_15_NO_3_P-H] at *m*/*z* 167 corresponding to choline phosphite. Finally, in the reaction samples containing IPF and ethanolamine in the non-aqueous solvents, the key peaks identified were as follows: [C_2_H_8_NO_4_P-H] at *m*/*z* 140 corresponding to phosphoethanolamine and finally [C_2_H_8_NO_3_P-H] at *m*/*z* 124 corresponding to ethanolamine phosphite. 

**Figure 2:**
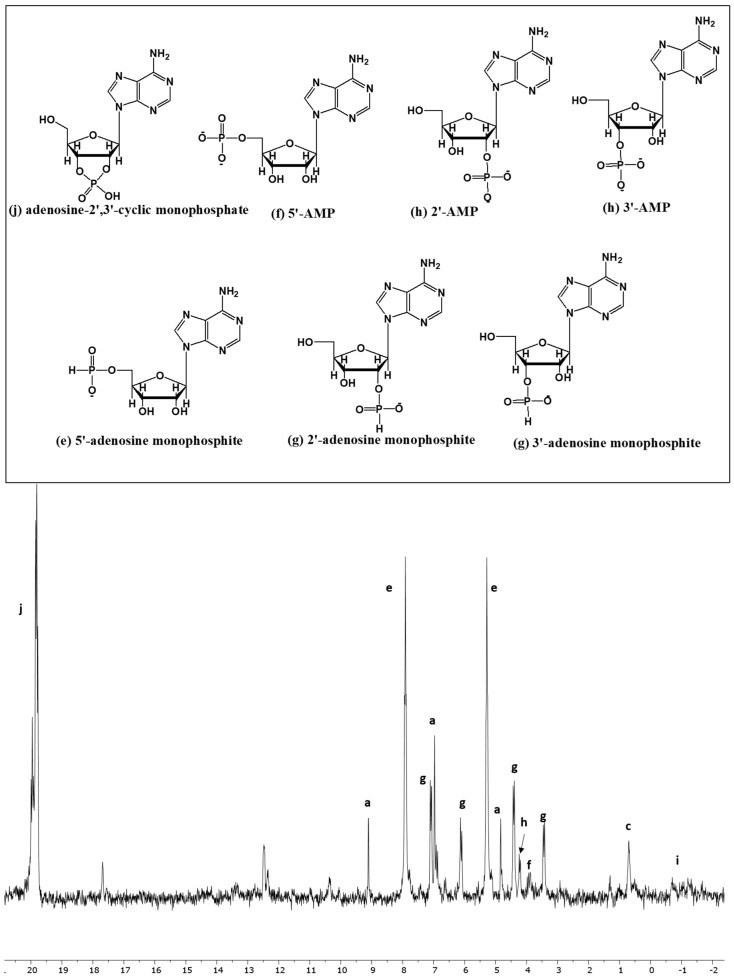
Heating reaction of IPF with adenosine in UAFW solvent for 3 days (Reaction sample No: AD-UAFW-3).

Various organophosphites were identified and characterized by analyzing their chemical shift values and C-O-P (carbon, oxygen, and phosphorus), as well as P-H (phosphorus and hydrogen), interactions of various organophosphorus species [39,48]. In the case of adenosine nucleoside, the best reaction sample was when the adenosine and IPF solution mixture was heated at 65–68 °C for 3 days and the solvent was UAFW. It produced about 89% of the phosphorylated and phosphonylated derivatives of adenosine. The compound adenosine-2′,3′-cyclic monophosphate (Figure 2, peak j) appeared as a multiplet around 20 ppm. The other cyclic derivative, i.e., adenosine-3′,5′-cyclic monophosphate derivative, was not present in the sample, which is usually located close to -2 ppm and appears as a doublet [48]. It should be noted that there seemed to be two sets of peaks around 20 ppm; apart from compound j (adenosine-2′,3′-cyclic monophosphate), there was possibly a double phosphorylation compound (2′,3′-cyclic, 5′-monophosphate or phosphite NMP). This observation was also consistent with other nucleosides, including uridine and cytidine, as well as our previous observations [48].

In the case of adenosine-5′- monophosphite (Figure 2, peaks labeled as e), H-coupling of ^31^P-NMR showed the splitting of this compound into a doublet of triplets with one between 7.5 and 8.0 ppm and the other 5.0 to 5.5 ppm. This also indicated the presence of a CH_2_-O-P bond, implying that the phosphite was attached at the 5′-carbon. The phosphonylated derivatives such as adenosine 2′- or 3′-monophosphites (peak g) appeared as doublets, showing the presence of a phosphite group via a CH-O-P bond. In the particular case of 2′ or 3′ monophosphite, the H-coupling of ^31^P-NMR showed the splitting of this compound into two doublets. Based on our previous observations the phosphonylated derivatives of organic compounds are usually located downfield of 5.0 ppm. The organophosphates (phosphate esters) are usually present between 2 and 5.5 ppm. The H-coupled splitting of ^31^P-NMR for adenosine 5′-monophosphate (peak f) appeared to be as a small triplet around 3.4 to 4.0 ppm, indicating the presence of a CH_2_-O-P type compound, and doublets (peak h) can represent 2′ or 3′-AMP, representing a CH-O-P type linkage, while peak i represents dimer (adenosine-phosphate-adenosine species) as reported previously [48] and it is generally located around −1 to −2 ppm. The H-coupling of ^31^P-NMR of the unreacted hypophosphite showed a splitting into a large triplet. Inorganic phosphite showed a splitting into a doublet in the H-coupled ^31^P-NMR spectrum and was also confirmed by calculating the coupling constant (~560 Hz). Inorganic condensed P compounds such as pyrophosphate appeared as a singlet peak in the H-coupled ^31^P-NMR spectrum, usually around −6 to −8 ppm. 

It is important to mention here that the peaks could easily be shifted right or left due to pH changes [52]. This is why each peak was identified by carefully looking at the splitting pattern in the H-coupled ^31^P-NMR spectrum, chemical shift values, and coupling constant (J). The samples were also spiked with standards whenever possible. Furthermore, finding the molecular weights of the targeted compounds via MS confirms some of these IDs. In case of adenosine, the compounds adenosine-5′-monophosphate and adenosine-2′,3′-cyclic monophosphate were confirmed by spiking with standards.

**Figure 3:**
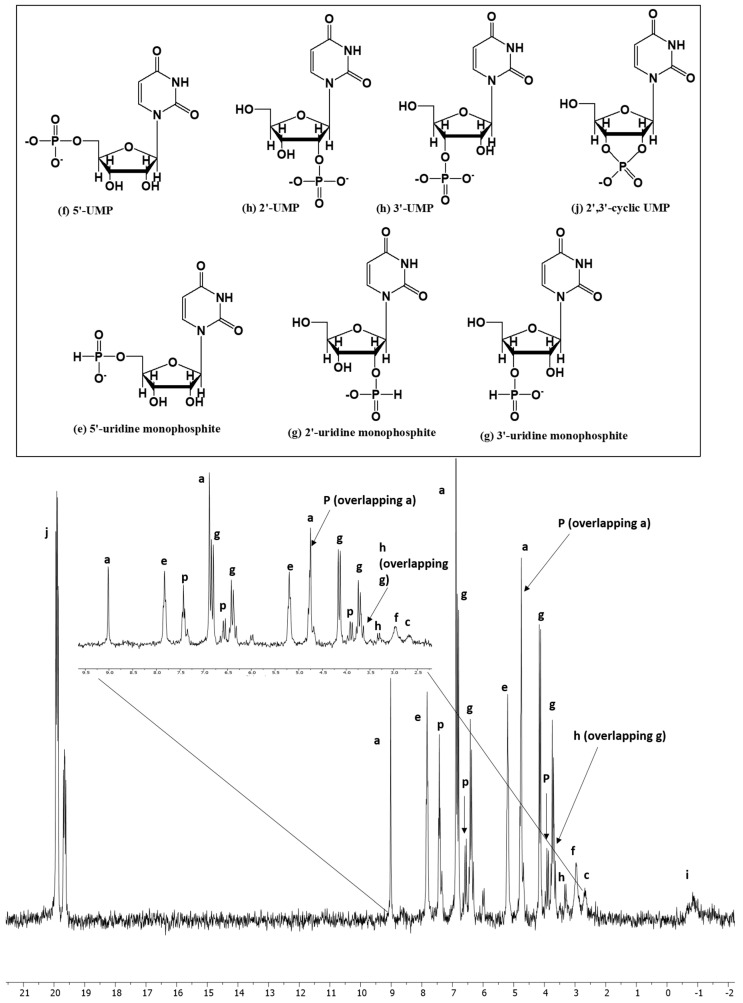
Uridine nucleoside reaction with IPF solution in UAFW solvent (Reaction UR-UAFW-2).

Uridine also readily reacted with the IPF solution. The relative abundances and products distributions (%) (based on the total dissolved P in the solution) were around 89%, when the reaction mixture was heated in the UAFW for 2 days (Table 1, reaction Sample no UR-UAFW-2). As described for adenosine, the peaks were identified by looking at the splitting pattern in the H-coupled ^31^P-NMR spectrum (Figure 3). Various organic (uridine) diphosphite in case of were also reported in our previous studies [39]. Overall, uridine nucleoside required a lower temperature window, i.e., 55–58 °C, for better reactivity. Percent fractions of organophosphorus compounds in the case of the three nucleosides are given in Table 3 based on ^31^P NMR integrations.

**Figure 4:**
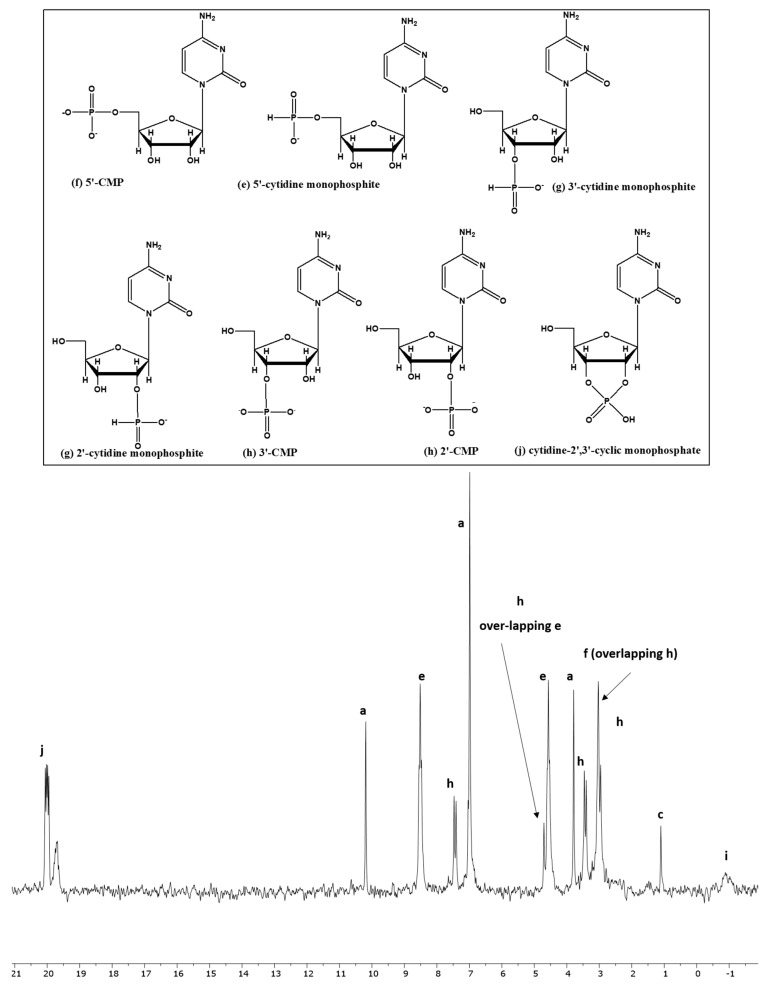
Reaction of cytidine nucleoside with IPF solution in UAFW (reaction sample CY-UAFW-2).

In the case of cytidine, the best reaction results (83% yields) were obtained when this nucleoside was heated with IPF solution (unsealed) at 65–68 °C, in the presence of UAFW as solvent. As mentioned above and as the case with uridine and adenosine, various phosphonylated and phosphorylated derivatives were identified by looking at the peak-splitting patterns (singlet, doublet, triplet, or multiplet) in the H-coupled spectrum of ^31^P-NMR peak locations, and spiking with standard compounds was done wherever the standards were available (Figure 4) [39,48,49,50]. 

**Figure 5:**
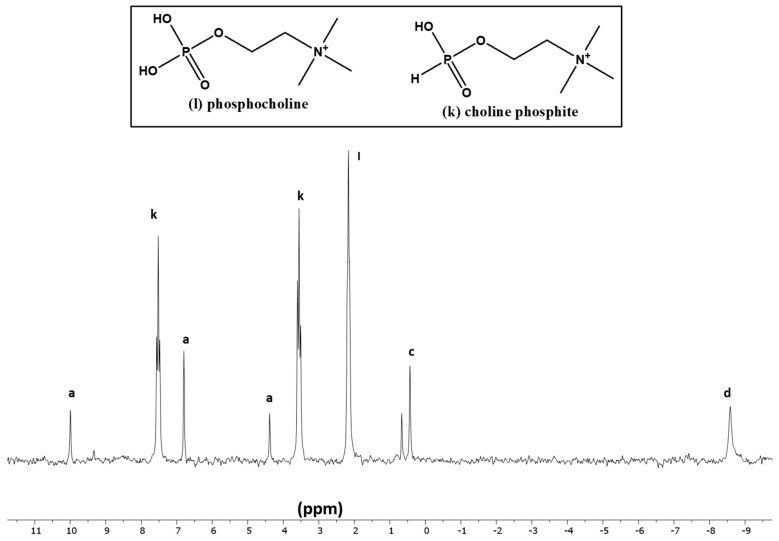
Reaction of choline chloride with IPF solution in UAFW (Reaction sample CH-UAFW-5).

**Figure 6:**
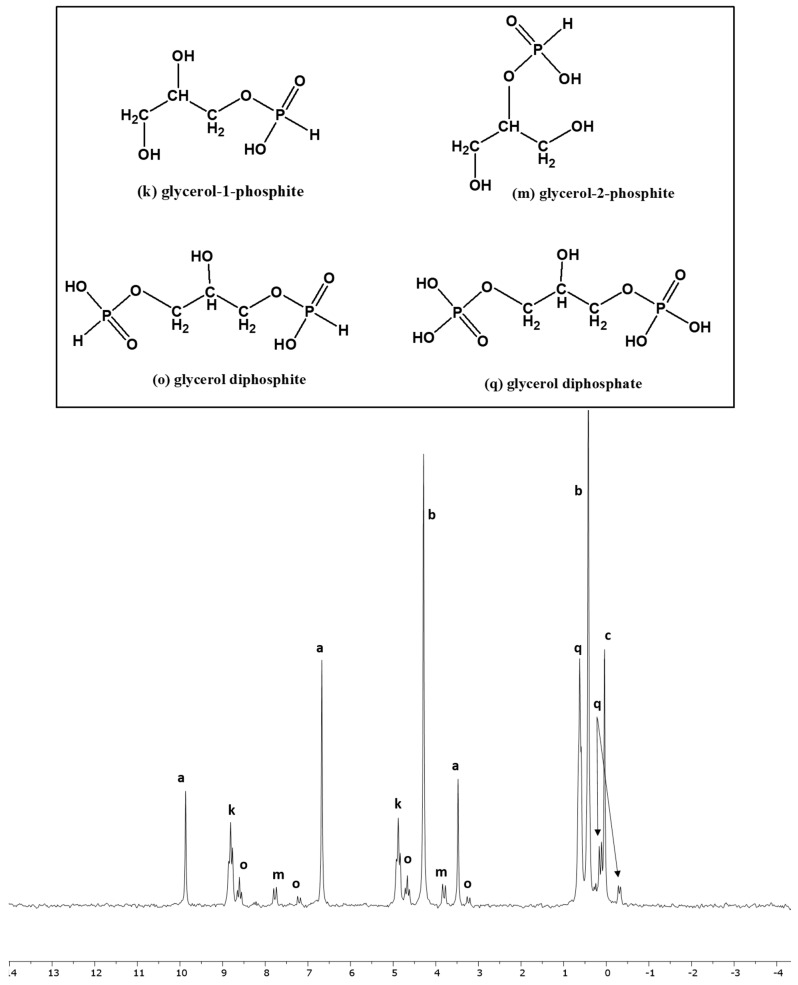
Reaction of glycerol with IPF solution in formamide (Reaction GL-Form-5).

In the case of both choline chloride and glycerol (Table 4, Figure 5 and Figure 6), the best reactions were observed at 65–67 °C in the presence of UAFW as a solvent. Choline chloride is a tertiary amine, and it has only one location for phosphorylation or phosphonylation. In this case, two triplets around 3.5 ppm and 7.5 ppm represented choline phosphite, while a large triplet around 2 to 3 ppm suggested the presence of phosphocholine (Figure 5). For glycerol, both solvents showed similar results. Nevertheless, UAFW was a still-better solvent with a higher fraction of organic-P at 50%. Usually, glycerol-1-phosphate appears as a triplet between 3 to 5.5 ppm, indicating the presence of a CH_2_-O-P bond in the H-coupled spectrum of ^31^P-NMR. Similarly, glycerol-2-phosphate appears as a doublet, generally preceding glcyerol-1-phosphate location, viz [22,29,50]. However, in both samples, both of these phosphorylated species were not detected. The phosphite derivatives of glycerol showed splitting into two triplets (glycerol-1-phosphite) and two doublets (glycerol-2-phosphite). The glycerol diphosphite or diphosphate (not pyrophosphate or pyrophosphite but phosphite/phosphate tied to different carbons on the glycerol molecule) were also identified by looking at the peak-splitting patterns in the H-coupled ^31^P-NMR. These compounds were also compared with our previous results [22,29,39,50].

**Figure 7:**
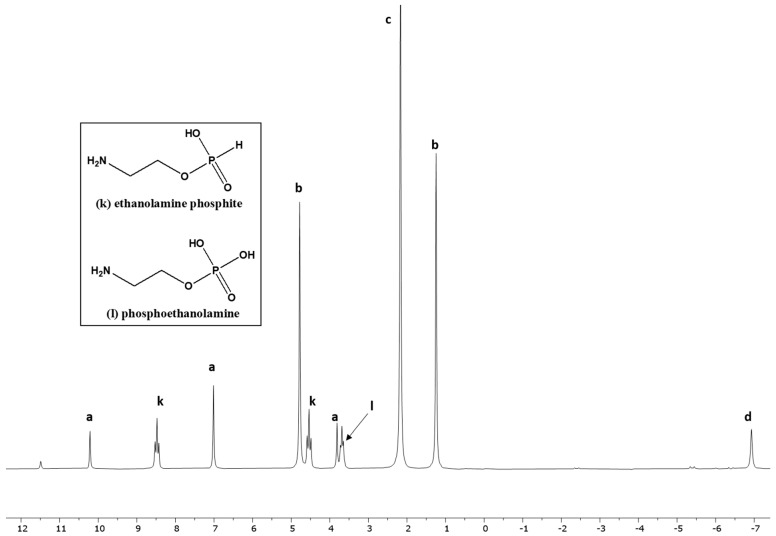
Reaction of ethanolamine with IPF solution in formamide (Reaction EA-UAFW-4).

In the case of ethanolamine, the best results were seen when it was heated with IPF at 55–57 °C in UAFW (Figure 7) (Sample EA-2, Table 1). In this case, the preferred site for phosphorylation or phosphonylation was the –OH group as compared to the –NH_2_ group (Figure 7).

Some reaction sets were also quantified by using an internal standard, which was 0.1 M PAA as described previously [48]. Each reaction was studied in UAFW. These yields are, with respect to the total phosphorus, added to the solution, which was the limiting reagent compared to the nucleoside substrate. In the case of adenosine, the yields were as follows: 2′-AMP and 3′-AMP combined yields (1.5%), 5′-AMP (0.5%), 2′,3′-cyclic AMP (12%), adenosine-phosphate-adenosine (A-P-A) (2%), 2′and 3′-adenosine-monophosphite (7.5%), and 5′-adenosine-monophosphite (11.8%), with a total yield of adenosine phosphites and phosphates to be around 35%, respectively. In the case of the uridine reaction in the UAFW, the best yields were as follows: 2′-UMP and 3′-UMP (combined yields 1%), 5′-UMP (0.5%), 2′,3′-cyclic UMP (11%), uridine-phosphate-uridine (U-P-U) (1.5%), 2′and 3′-uridine-monophosphite (8%), and 5′-uridine-monophosphite (14%), with a total yield of uridine phosphites and phosphates to be around 36%. Similarly, for cytidine, the best reaction yields were as follows: 2′-CMP and 3′-CMP (combined yields 0.5%), 5′-CMP (2%), 2′,3′-cyclic CMP (7%), cytidine-phosphate-cytidine (C-P-C) (1.5%), 2′and 3′-cytidine-monophosphite combined yields (7.5%), and 5′-cytidine-monophosphite (10%), with a total yield of cytidine phosphites and phosphates to be around 28.5% (SI, and also see ref. [48]). 

In the case of other organics including glycerol, ethanolamine and choline chloride, the best reaction (in UAFW) yields based on the internal standard (PAA) were also calculated. For glycerol, the yields were as follows: glycerol-1-phosphate (13%), glycerol-1-phosphite (16%), glycerol-2-phosphite (1%), glycerol-diphosphite species (2.5%), and glycerol-phosphate-glycerol (1%). This was a total of both glycerol phosphates and phosphites of around 33.5%. In the case of choline chloride, the yields were as follows: choline phosphite (18.54%), phosphocholine (17.5%) (total for ethanolamine was around 36%), and finally, in the case of ethanolamine, the yields were as follows: ethanolamine phosphite (35%) and phosphoethanolamine (16%), with a total of both phosphites and phosphates of ethanolamine of around 51%.

In a separate set of experiments, we also studied the release of prebiotically relevant phosphate and phosphite materials and the possible release of soluble phosphorus from these mineral sources at 65–68 °C for three days under sealed conditions (Table 5). These studies were carried out in the non-aqueous and semi-aqueous solvents (formamide and UAFW) used in the phosphorylation and phosphonylation studies. The extent of the release of soluble phosphorus was determined on the basis of the total molarity of [P] in the solution as previously [37,51]. The best result was observed, with total molarity of the P in the solution, to be around 0.1 M. We also observed the generation of pyrophosphite in this case. Overall, both formamide and UAFW showed an affinity for P solubilization. However, no P signals in the ^31^P-NMR were observed in either phosphate minerals vivianite or apatite, at least not under same set of temperature, solvent volume, and, most importantly, number of scans for the ^31^P-NMR. The number of scans in the case of natural samples, particularly minerals, was from 5000 to 10,000 per sample. Both phosphite materials actively released P into the solvent, indicating the ease of P-release.

## 4. Discussion

**Figure 8:**
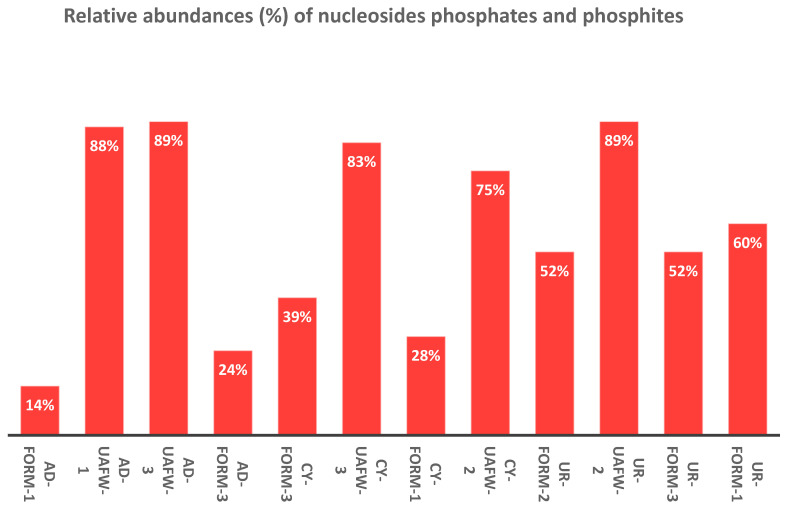
Comparative fractions (%) of various nucleoside phosphates and phosphites as a function of total P integrations.

**Figure 9:**
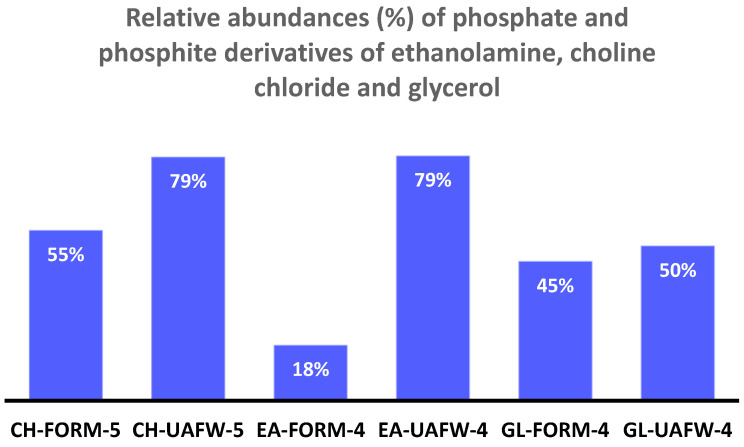
Comparative fractions (%) of various organic phosphates and phosphites as a function of total P integrations. These abundances were based on the amount of dissolved P in the solution and the peak integration method.

Heating organics with the oxidation products of hypophosphite, generated via Fenton reaction in non-aqueous and semi-aqueous solvents, formed phosphate and phosphite esters of prebiotic relevance. The reactions happened under mild heating at 55–68 °C and did not require a catalyst, condensation agent, or other additive. Our two-way approach, of using (1) non-aqueous and semi-aqueous solvents and (2) a reduced P source, seemed to be quite effective in forming various organophosphorus compounds with ease. In contrast to our previous studies, urea was not required [48]. However, the better efficiency of the UAFW solvent over formamide can also be attributed to urea being part of the solvent composition. The overall yields in the current studies ranged from 14 to 89% (based on the amount of dissolved P) (Figure 8 and Figure 9). We obtained a variety of phosphorylated and phosphonylated derivatives of nucleosides (uridine, adenosine, and cytidine) and other organics including glycerol, ethanolamine, and choline chloride. This overall increase in the reactivity of inorganic-P molecules generated via Fenton chemistry with the organics suggests that the reduced P compounds and their oxidation products bear an increased reactivity compared to their phosphate counterparts, and could have played a role in the origin of biological P compounds on the early Earth.

Furthermore, heating reactions of IPF with nucleosides in the anhydrous solvents also form phosphodiesters of uridine, adenosine, and cytidine. At present, the exact structure of the dimer species is not clear; however, based on our previous studies, we suggest that these diester species were likely formed via the opening of 2′,3′-cyclic monophosphate. This is supported by an observation in our previous study, in which heating an IPF solution containing 2′-deoxyadenosine and urea at 55–60 °C, leading to dryness, did not form any diester molecules, implying that diester formation is linked with the ring opening of the nucleoside-2′,3′-cyclic monophosphate [48]. We have also reported the generation of diester compounds of uridine by the heating reactions of uridine with pyrophosphate in the presence of Mg^2+^ and urea [49]. In both previous studies, diesters appear only when cyclic monoester is formed [48,49]. 

The present work suggests the prebiotic syntheses of a variety of molecules including nucleotides of uridine, adenosine, and cytidine, and their respective phosphate diesters that play a significant role in biochemistry. These diester molecules serve as a molecular ‘tape’ that connects the individual nucleotides in DNA and RNA through a sugar–phosphate backbone [49,53,54]. Also, the other essential phosphate esters, such as glycerol phosphate, phosphoethanolamine, and phosphocholine, are also found in living organisms’ biochemistry, especially in cell membranes. Modern life needs these phosphorylated biomolecules for storing genetic information, cell structures, respiration, and many other functions. 

Phosphorylation on the early Earth would have played a key role in the chemical milieu, forming phosphorylated biomolecules essential to life through the oxidation of reduced P compounds such as hypophosphite. Hypophosphite and related species such as H-phosphinic acid (H_3_PO_2_) can also be sourced from meteoritic mineral schreibersite [37,55], and hence can be regarded as a prebiotic source of P present on the early Earth. Besides a reduced P source, the proposed reactions would also need an environment with both soluble iron and reduced P compounds, which is prebiotically plausbile [51]. 

Moreover, the semi-aqueous solvent (UAFW) in the study can be prebiotic. Urea has been prebiotically synthesized in the classic Urey–Miller gas-discharge experiments [56], by exposing ammonium cyanide to sunlight [57], and it is also identified to be a hydrolysis product of cyanamide [58], while ammonium formate is a hydrolysis product of HCN [59]. Therefore, both of these compounds can be considered to be essentially prebiotic [60,61]. Research has shown that, on heating, the UAFW eutectic solvent mixture is partially converted into formamide, therefore forming a four-component solvent mixture. This UAFW solvent system promotes dehydration to support C-O-P bond formation through condensation [30]. Similarly, formamide is also considered to be a prebiotically significant compound, and has been employed in demonstrating various prebiotic chemical reactions for decades [14,15,19,20,21,22,23,24,25]. 

This discussion supports the idea of the plausibility of ‘a warm drying alkaline pond’ on the early Earth with dissolved Fe^2+^, reduced P compounds (either supplied by meteorites [37] or formed through Fenton chemistry [41]), and other components forming mixtures with water such as ammonium formate, urea, and even formamide. 

Another important ingredient to support Fenton reactions on the prebiotic Earth would be the H_2_O_2_ that would possibly have been supplied to ‘Snowball Earth’. Such events would potentially result in a relatively weak hydrological cycle that would have sustained the formation of H_2_O_2,_ when coupled with certain photochemical reactions of water or ice [42]. Furthermore, Fenton reactions have also been suggested on the Martian surface [62], and the idea is further supported by the discovery of H_2_O_2_ on the Martian surface [63,64].

**Figure 10:**
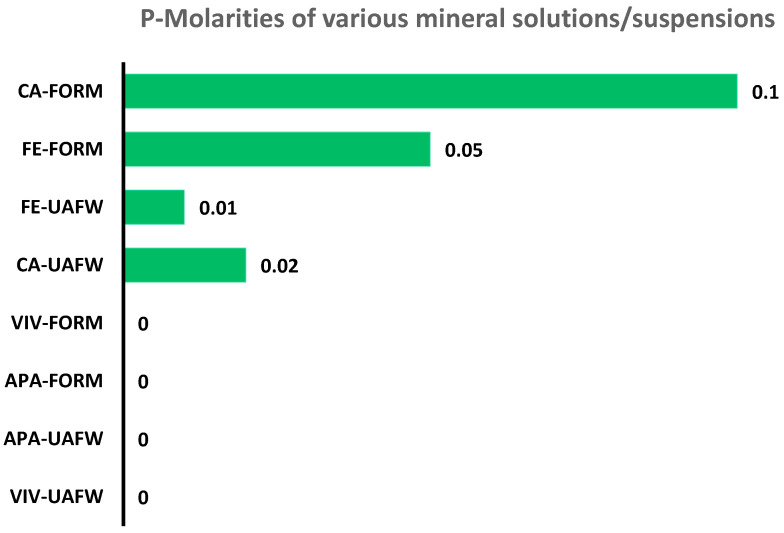
Phosphorus release from various minerals including vivianite, iron phosphite, apatite and calcium phosphite in formamide and UAFW solvents at 65–68 °C (3 days). The molarity of P in each solution was calculated by using Equation 1. Each value represents molarity [M]. In the present study, calcium phosphite in formamide seemed to have released most amount of P into the solution. Abbreviations are as follows: VIV (vivianite), CA (calcium phosphite), FE (iron phosphite), APA (apatite), Form (formamide), and UAFW (urea, ammonium formate, and water).

Our experiments studying the release of P from various P minerals, including vivianite, apatite, and phosphites of calcium and iron, showed that under similar conditions (e.g., sealed heating reactions, 65–68 °C, 3 days, formamide or UAFW), only the phosphite materials released P. Interestingly, we also observed the formation of condensed P compounds (pyrophosphite) while studying the release of P from a solution of CaHPO_3_. Overall, CaHPO_3_ seemed to work out best in releasing the P into the solution of formamide under the reported experimental conditions (Figure 10). We detected no P signals in the ^31^P-NMR from either of the phosphate minerals.

Considering that ancient oceans were anoxic and Fe (II)-rich [65,66,67,68], it would seem highly plausible for the phosphate and phosphite mineral phases of iron to be precipiated out in the early oceans in the form of viviante (for phosphate) and FeHPO_3_ (for phosphite). Apatite [Ca_5_(PO_4_)_3_(F,Cl,OH)] is considered to be the dominant form of phosphates on the early Earth (and other elements) [4,69], but if the early oceans were slightly less alkaline than today’s oceans, likely due to a higher partial CO_2_ pressure [3,70], then the precipitation of acid calcium salts of phosphate [71] and even phosphite could have been possible in early oceans.

Our results show the significance and increased reactivity of reduced (inorganic) P compounds towards organic compounds, and how these reduced P compounds, even tied up as minerals, could potentially have readily released phosphite into the early oceans to facilitate the origin of biological P compounds on the early Earth.

## 5. Conclusions

Phosphate and phosphite derivatives of various nucleosides (adenosine, uridine, and cytidine), alcohols (glycerol and ethanolamine), and organic ammonium compound (choline chloride) were prepared by using a reduced P source that was obtained from a Fenton reaction of hypophosphite [48]. The phosphorylation and phosphonylation reactions were carried out at 55–68 ^o^C from 20 h to 5 days, unsealed in a non-aqueous solvent (formamide) and a semi-aqueous solvent (UAFW). In our studies, UAFW seemed to be a better reaction medium than formamide. Also, urea was not found necessary in our current studies as non-aqueous solvents seemed to support the overall condensation process.

The release of soluble P (as phosphite and phosphate) was also studied at 65–68 °C under sealed conditions and with consistent stirring. It was seen that under the reported conditions, the phosphate minerals vivianite (Fe_3_(PO_4_)_2_·8H_2_O), and apatite (Ca_5_(PO_4_)_3_(F, Cl, OH)) did not release any P into the solution (as indicated by the detection of no signal in the ^31^P-NMR), while their phosphite compounds, iron(II) phosphite (FeHPO_3_) and calcium phosphite (CaHPO_3_), not only released phosphite into the solution but also formed a high-energy condensed phosphite, i.e., pyrophosphite (at 65–68 °C under sealed conditions). This indicated that reduced P compounds could have played a significant role in the origin of biological-P compounds on the early Earth.

## Figures and Tables

**Table 1:** Reaction conditions of various reaction samples.

Sample Name	Description
For	7 mL IPF solution, 4 mL formamide, pH = 11–12, 65–68 °C, 3 days
UAFW	7 mL IPF solution, 4 mL UAFW, pH = 11, 65–68 °C, 3 days
AD-Form-1	0.65 g adenosine, 7 mL IPF solution, 4 mL formamide, 65–68 °C, 1 day
AD-UAFW-1	0.65 g adenosine, 7 mL IPF solution, 4 mL UAFW, 65–68 °C, 1 day
AD-UAFW3	0.65 g adenosine, 7 mL IPF solution, 4 mL UAFW, 65–68 °C, 3 days
AD-Form-3	0.65 g adenosine, 7 mL IPF solution, 4 mL formamide, 65–68 °C, 3 days
CY-Form-3	0.60 g cytidine, 7 mL IPF solution, 4 mL formamide, 65–67 °C, 3 days
CY-UAFW-3	0.60 g cytidine, 7 mL IPF solution, 4 mL UAFW, 65–67 °C, 3 days
CY-Form-1	0.60 g cytidine, 7 mL IPF solution, 4 mL formamide, 65–67 °C, 1 day
CY-UAFW-2	0.60 g cytidine, 7 mL IPF solution, 4 mL UAFW, 65–67 °C, 2 days
UR-Form-2	0.65 g uridine, 7 mL IPF solution, 4 mL formamide, 55–57 °C, 2 days
UR-UAFW-2	0.65 g uridine, 7 mL IPF solution, 4 mL UAFW, 55–57 °C, 2 days
UR-Form-3	0.65 g uridine, 7 mL IPF solution, 4 mL formamide, 55–58 °C, 3 days
UR-Form-1	0.65 g uridine, 7 mL IPF solution, 4 mL UAFW, 55–57 °C, 1 day
CH-Form-5	0.80 g choline chloride, 7 mL IPF solution, 4 mL formamide, 65–68 °C, 5 days
CH-UAFW-5	0.80 g choline chloride, 7 mL IPF solution, 4 mL UAFW, 65–68 °C, 5 days
EA-Form-4	0.70 g ethanolamine, 7 mL IPF solution, 4 mL formamide, 55–57 °C, 4 days
EA-UAFW-4	0.70 g ethanolamine, 7 mL IPF solution, 4 mL UAFW, 55–57 °C, 4 days
GL-Form-4	0.80 g glycerol, 7 mL IPF solution, 4 mL formamide, 65–68 °C, 4 days
GL-UAFW-4	0.75 g glycerol, 7 mL IPF solution, 4 mL UAFW, 65–68 °C, 4 days

Prebiotic synthesis of organic P esters of biological significance. Various conditions attempted in the study. Each of the samples was heated unsealed at 55–68 °C for 1 to 5 days on a hot plate under the fume hood. The pH of each solution was around 10–11. No additive/catalysts were used. Where UAFW stands for urea: ammonium formate: water, IPF solution means inorganic P Fenton solution, and Form represents formamide. Also, the abbreviations for the organic compounds are as follows: AD (adenosine), UR (uridine), CY (cytidine), GL (glycerol), CH (choline chloride), and EA (ethanolamine). Reaction samples were heated unsealed for a given amount of time mimicking a ‘Warm-Pool Model’ Theme. Various numbers with the labeled names of the samples represent the days of heating.

**Table 2:** Reaction conditions of various reaction samples to study the release of P from various phosphorus minerals into non-aqueous solvents.

Sample	Description
	Reaction Set-1
FE-Form	0.200 g FeHPO_3_, 4mL formamide, pH = 8, 65–68 °C
CA-Form	0.200 g CaHPO_3_, 4 mL formamide, pH = 6.5, 65–68 °C,
APA-Form	0.200 g apatite (Ca_5_(PO_4_)_3_(F, Cl, OH), 4 mL formamide, pH = 8, 65–68 °C
VIV-Form	0.200 g vivianite (Fe_3_(PO_4_)_2_·8H_2_O), 4 mL formamide, pH = 8, 65–68 °C
	Reaction Set-2
FE-UAFW	0.200 g FeHPO_3_, 4 mL UAFW, pH = 6, 65–68 °C
CA-UAFW	0.200 g CaHPO_3_, 4 mL UAFW, pH = 5–6, 65–68 °C,
APA-UAFW	0.200 g apatite (Ca_5_(PO_4_)_3_(F, Cl, OH), 4 mL UAFW, pH = 5, 65–68 °C
VIV-UAFW	0.200 g vivianite (Fe_3_(PO_4_)_2_·8H_2_O), 4 mL UAFW, pH = 5, 65–68 °C

Each of the samples was heated under sealed conditions at 65–68 °C for 3 days. Two reaction sets of exact conditions were carried out, with the only difference being that Set-1 comprised of formamide while Set-2 contained UAFW. Also, various abbreviations are as follows: VIV (vivianite), CA (calcium phosphite), FE (iron phosphite), APA (apatite), Form (formamide), and UAFW (urea, ammonium formate, and water).

**Table 3:** The relative abundances (%) (with fractions relative to 100% total NMR integration) of various inorganic P products produced in the reactions comprising of nucleosides.

Sample Name	Hypophosphite	Phosphite	Orthophosphate	In. condensed P	5′-mono-PO_3_	5′-mono-PO_4_	2′-+ 3′-mono-PO_3_	2′-+ 3′-mono-PO_4_	Dimer Species	2′, 3′-cyc. Org. PO_4_	Nucleoside Diphosphite	Total Org. PO_4_	Total Org. PO_3_	^T^C-O-P
	**a**	**b**	**c**	**d**	**e**	**f**	**g**	**h**	**i**	**j**	**p**			
Ad-Form-1	18	39	11	18	3	ND	ND	ND	8	3	ND	11	3	14
Ad-UAFW-1	8	ND	4.5	ND	28	1.5	19	6	ND	33	ND	40.5	47	87.5
Ad-UAFW-3	8	ND	3	ND	32	2	16	6	ND	33	ND	41	48	89
Ad-form-3	15	35	16	10	11	8	ND	ND	ND	5	ND	12	11	24
Cy-form-3	15	38	8	ND	8	2	3	2	8	16	ND	28	11	39
Cy-UAFW-3	10	ND	7	ND	30	ND	23	ND	7	23	ND	30	53	83
Cy-Form-1	13	44	13	ND	5	12	3	6	ND	2	ND	20	8	28
Cy-UAFW-2	18	6	1	ND	23	10	27	ND	2	13	ND	23	50	75
Ur-Form-2	20	26	7	ND	12	15	11	4	3	7	ND	29	23	52
Ur-UAFW-2	10	ND	2	ND	15	5	24	9	4	23	8	41	47	89
Ur-Form-3	9	37	2	ND	23	2	12	ND	6	9	ND	17	35	52
Ur-Form-1	19	19	2	0.5	19	11	14	9	1	6	ND	27	33	60

The relative abundances (%) of the inorganic P products were calculated on the basis of the total P dissolved and by the peak integration method as in our previous studies [29,48,49,50]. The relative abundances can also be considered as yields (%) based on the total dissolved P in the given solution. This is due to the fact that the relaxation time for (^31^P-NMR analysis) between the two scans in some samples was increased from 2 s to 30 s. This relaxation time ensures the NMR is quantitative. It was found that peak integral values and relative abundances of various P species remained almost the same for each sample, whether the relaxation time was 2 or 30 s. Various conditions and the names and descriptions of the samples are explained in Table 1. ND means not detected, ^T^C-O-P means sum of both organic phosphites and organic phosphate species in a sample, and ‘d’ means total sum of all inorganic condensed P compounds detected.

**Table 4:** The relative abundances (%) of various inorganic P products containing organic compounds other than nucleosides such as glycerol, choline chloride, and ethanolamine.

Sample Name	Hypophosphite	Phosphite	Orthophosphate	In. Condensed P	Organic-1-PO_3_	Organic-1-PO_4_	Organic-2-PO_3_	Organic-2-PO_4_	Organic Diphosphite	Organic Diphosphate	Total Org. PO_4_	Total Org. PO_3_	^T^C-O-P
	**a**	**b**	**c**	**d**	**k**	**l**	**m**	**n**	**o**	**q**			
CH-Form-5	14	23	3	4	23	32	NA	NA	NA	NA	32	23	55
CH-UAFW-5	8	ND	5.3	8	47	31.6	ND	ND	ND	NA	31.6	47	78.6
EA-Form-4	8	51	19	4	12	6	NA	NA	NA	NA	6	12	18
EA-UAFW-4	18	ND	3	ND	53	26	NA	NA	NA	NA	26	53	79
GL-Form-4	14	35	7	ND	14	ND	3	ND	5	22	22	22	45
GL-UAFW-5	16	28	6	ND	37	ND	5	ND	ND	8	8	42	50

The relative abundances (%) of the inorganic P products were calculated on the basis of the total P dissolved and by the peak integration method as in our previous studies [29,48,49,50]. The relative abundances can also be considered as yields (%) based on the total dissolved P in the given solution (see caption for Table 3). Various conditions and the names and descriptions of the samples are explained in Table 1. ND means not detected, ^T^C-O-P means sum of both organic phosphites and organic phosphate species in a sample, and ‘d’ means total sum of all inorganic condensed P compounds detected. The species that are not possible for a particular compound are left with sign NA.

**Table 5:** Amounts of P released from various prebiotically relevant P minerals.

Sample	Phosphate (%)	Phosphite (%)	Pyrophophite (%)	[M]_T_
Fe-Form	47.17%	52.83%	BDL	0.05
VIV-UAFW	BDL	BDL	BDL	-
FE-UAFW	2.43%	97.57%	BDL	0.01
APA-UAFW	BDL	BDL	BDL	-
CA-UAFW	1.43%	98.57%	BDL	0.02
APA-Form	BDL	BDL	BDL	-
VIV-Form	BDL	BDL	BDL	-
CA-Form	1.34%	90.50%	8.15%	0.1

The relative abundances (%) of the inorganic P products were calculated on the basis of the total P dissolved and by the peak integration method as mentioned above [29,39,48,49,50,51]. The details of the samples are given in Table 2. Some of the abbreviation’s descriptions are as follows: BDL (below detection limit), [M]_T_ (total molarity of phosphite in the solution). Also, various abbreviations are as follows: VIV (vivianite), CA (calcium phosphite), FE (iron phosphite), APA (apatite), Form (formamide), and UAFW (urea, ammonium formate, and water). The total molarity of the solution was based on the relative abundance of the P species in each of the solutions with ~10% error factor.

## Data Availability

NMR raw files and all the other relevant research results can be obtained by request of the corresponding author.

## Supplementary Materials

The following supporting information can be downloaded at: https://www.mdpi.com/article/10.3390/life13112134/s1, Figures S1–S6: H-coupled and decoupled, ^31^P-NMR spectra of choline chloride, glycerol ethanolamine, uridine, cytidine, and adenosine; Figure S7: quantification of organophosphorus compounds with procedure.
