# Supplementary material for: Prebiotic Syntheses of Organophosphorus Compounds from Reduced Source of Phosphorus in Non-Aqueous Solvents
Source: Life (Basel). 2023 Oct 29;13(11):2134. doi: 10.3390/life13112134 (PMC10672063; doi:10.3390/life13112134)
Supplement: Supplementary file 1 [file life-13-02134-s001.zip › life-2664370-supplementary.pdf]

Supporting information

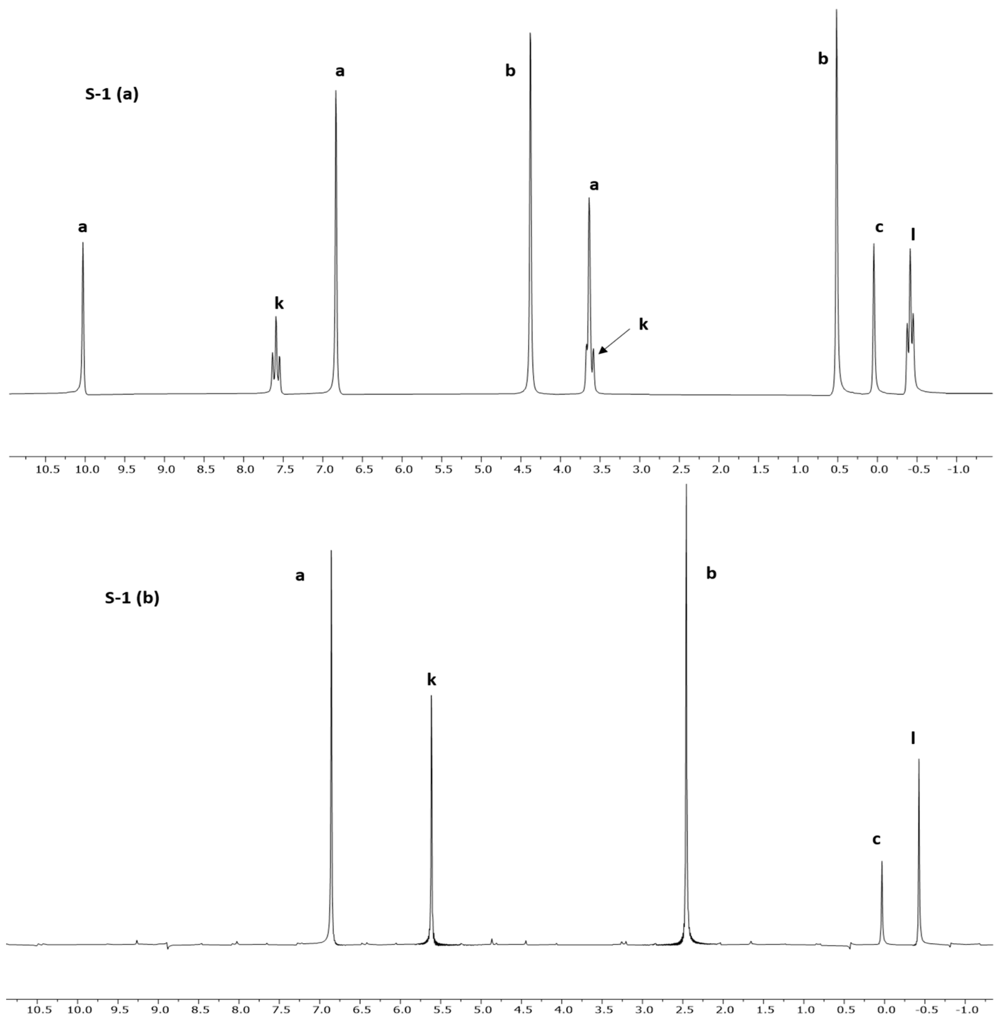

Figure S1. ^{31}P-NMR, H-coupled (S-1(a)) and decoupled (S-1 (b)) spectra of a reaction sample containing (0.80 g) choline chloride, (7 mL) IPF solution, (4 mL) UAFW, heated, unsealed at 65-68 °C for 20 hours.

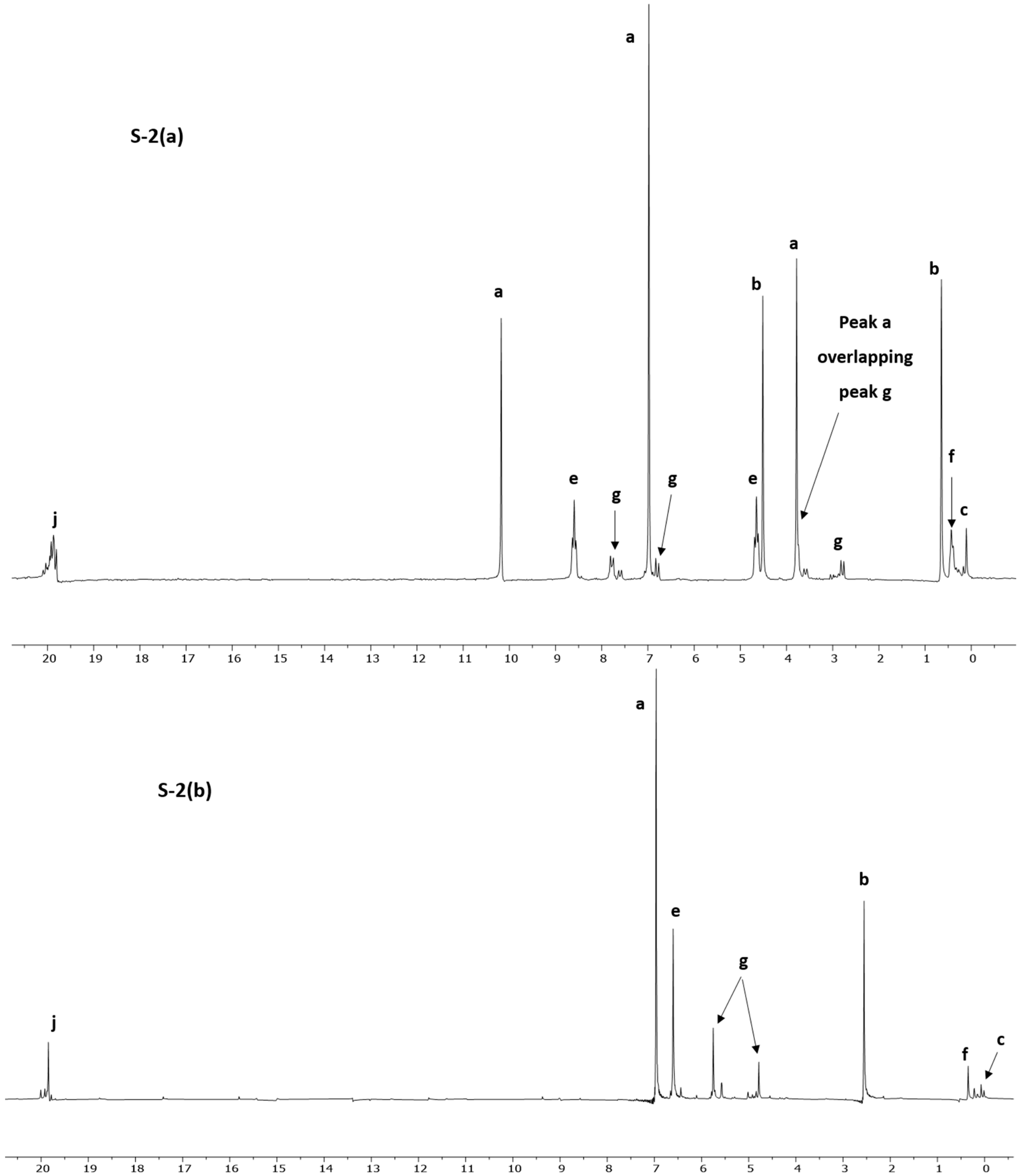

Figure S2. ^{31}P-NMR, H-coupled (S-2(a)) and decoupled (S-2 (b)) spectra of a reaction sample containing (0.65 g) adenosine, (7 mL) IPF solution, (4 mL) UAFW, heated, unsealed at 65-68 °C for 20 hours.

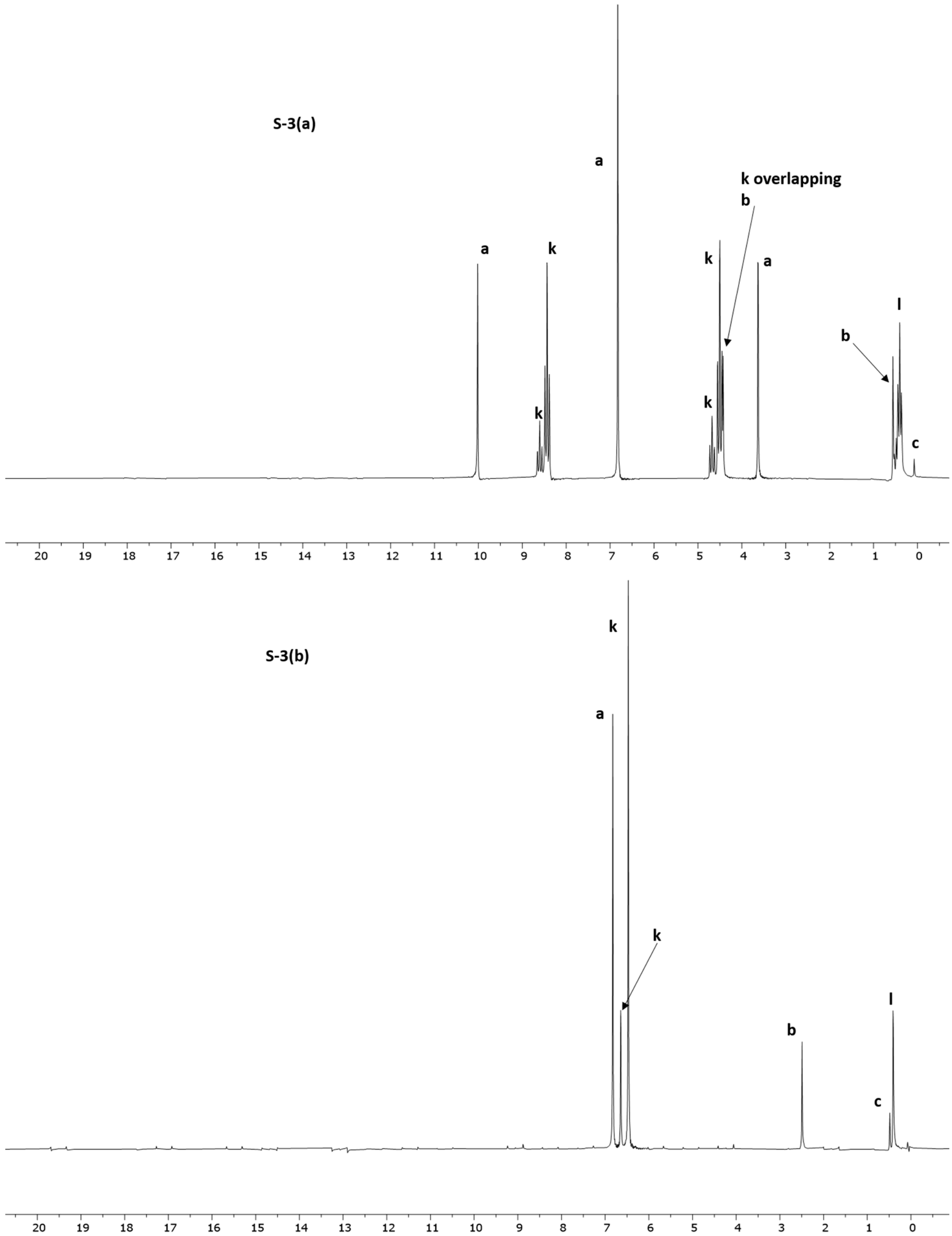

Figure S3. ^{31}P-NMR, H-coupled (S-3(a)) and decoupled (S-3 (b)) spectra of a reaction sample containing (0.70 g) ethanolamine, (7 mL) IPF solution, (4 mL) UAFW, heated, unsealed at 55-57 °C for 20 hours.Where the small peak k represents the reaction site involving NH_2 end of ethanolamine with the phosphite to form N-P bond. However, the preferential site still seemed to be the CH_2-OH of ethanolamine.

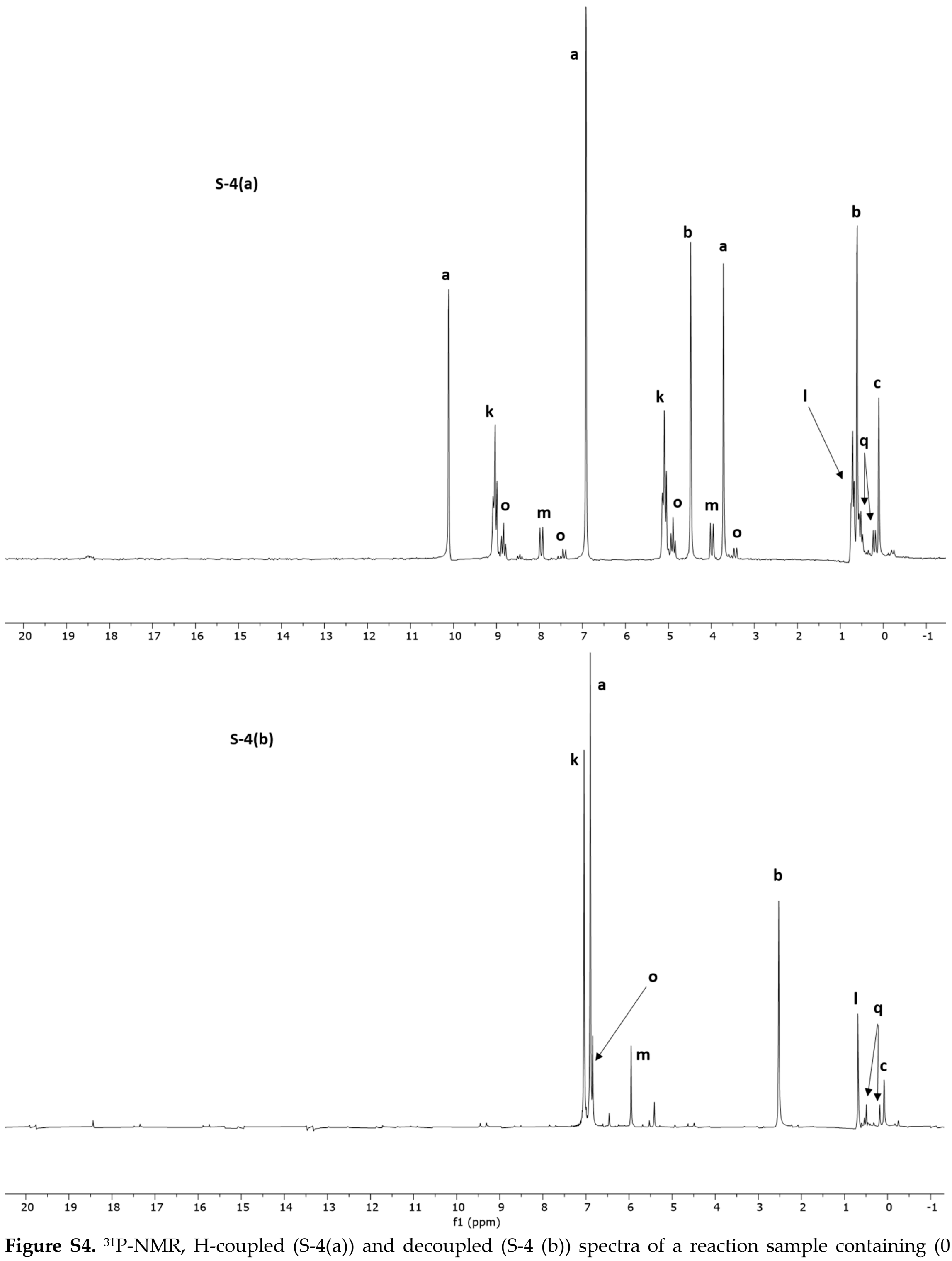

Figure S4. ^{31}P-NMR, H-coupled (S-4(a)) and decoupled (S-4 (b)) spectra of a reaction sample containing (0.70 g) glycerol, (7 mL) IPF solution, (4 mL) UAFW, heated, unsealed at 65-68 °C for 20 hours.

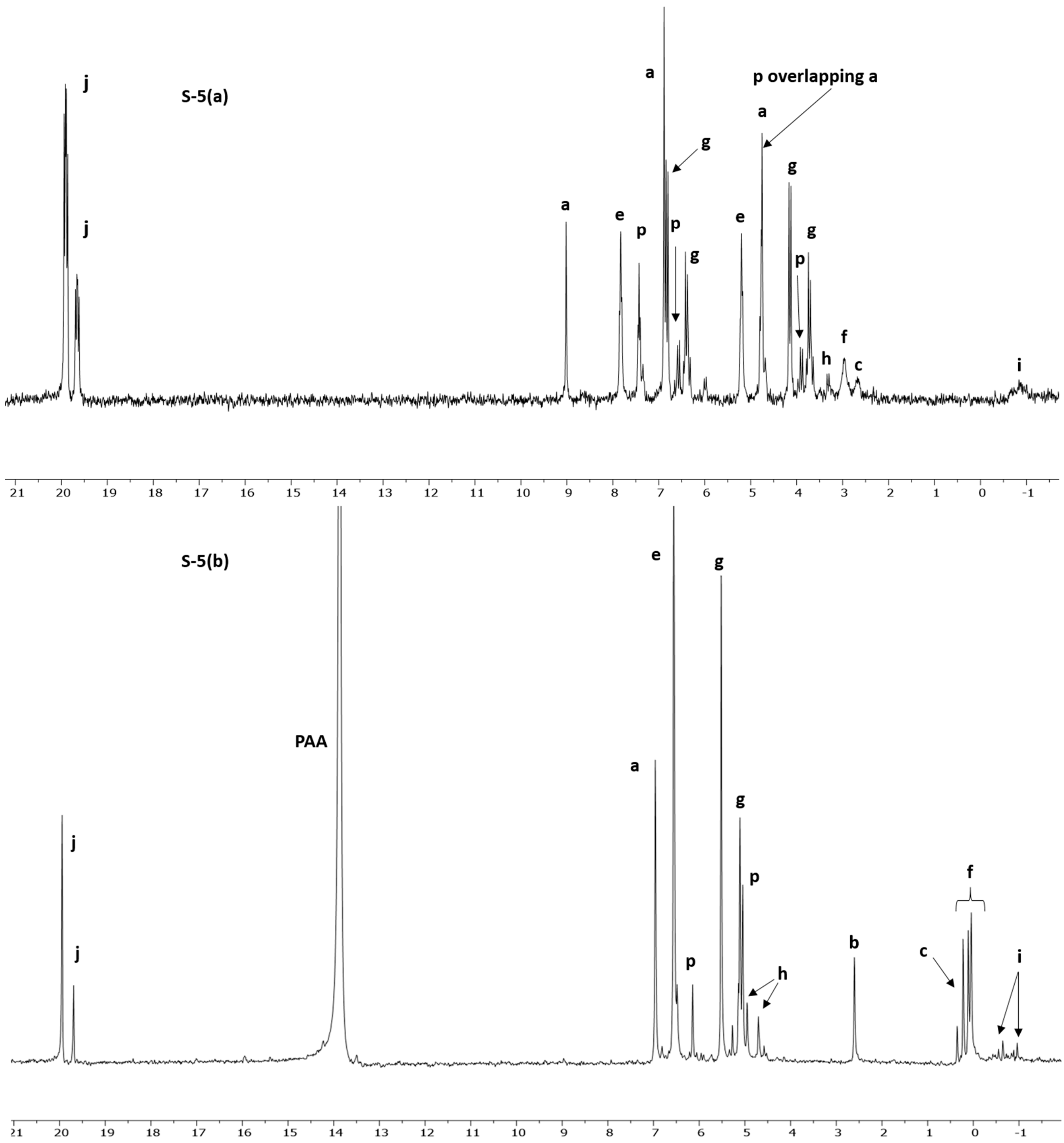

Figure S5. ^{31}P-NMR, H-coupled (S-5(a)) and decoupled (S-5(b)) spectra of a reaction sample containing uridine (reaction sample U-5, Table 1), IPF solution and UAFW.

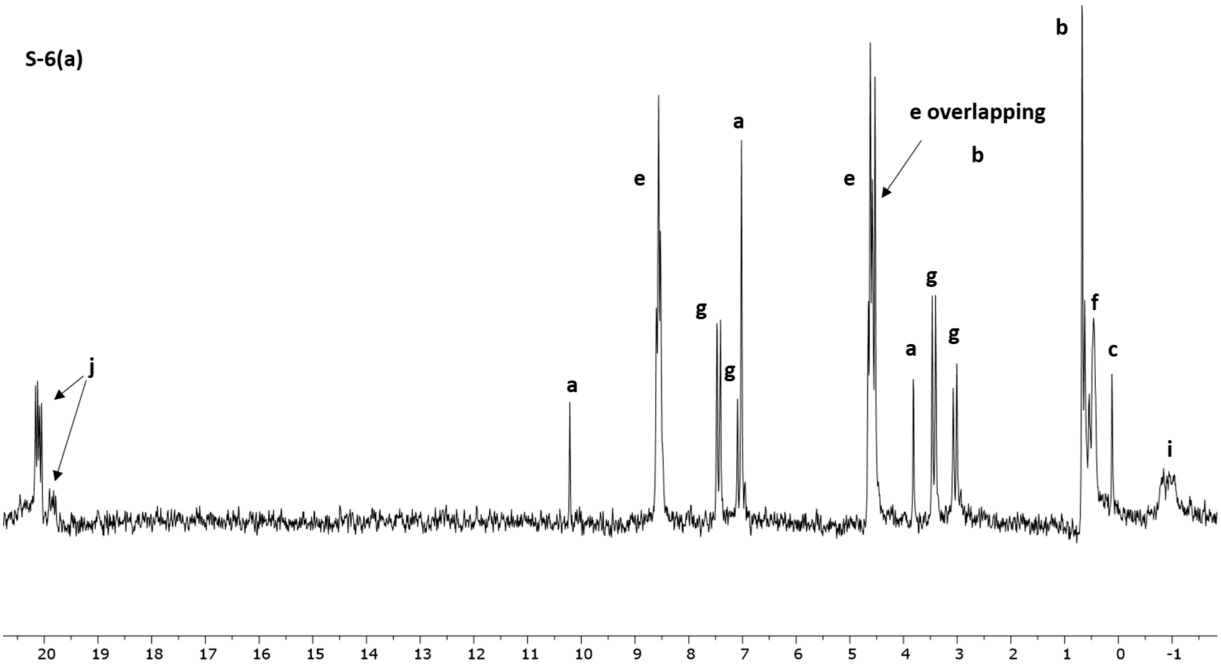

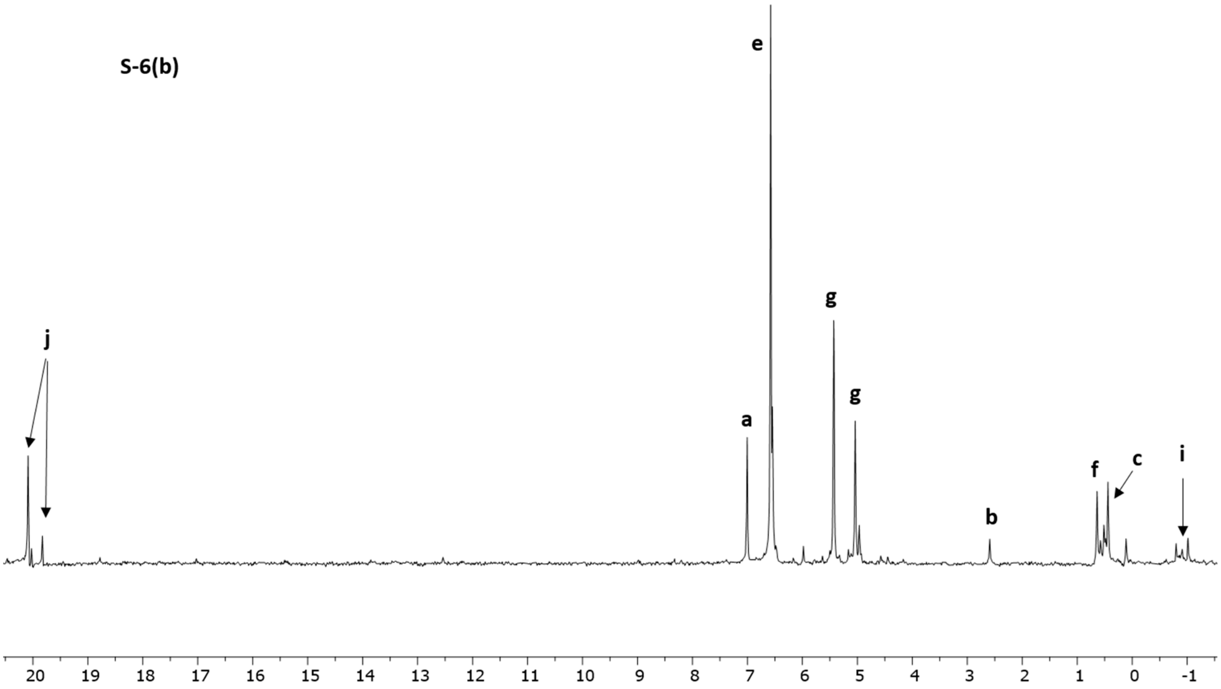

Figure S6. ^{31}P-NMR, H-coupled (S6(a)) and decoupled (S-6(b)) spectra of a reaction sample containing cytidine (reaction sample Cy-UAFW-3, Table 1), IPF solution and UAFW. Note that, this particular ^{31}P-NMR was studied after four years post phosphorylation/phosphonylation reactions in the UAFW. Where peak f, a triplet (CH_2-O-P) represents 5′-CMP (cytidine monophosphate) that is shown as a hydroylzed (or an oxidation) product of the reaction mixture that was stored at room temperature.

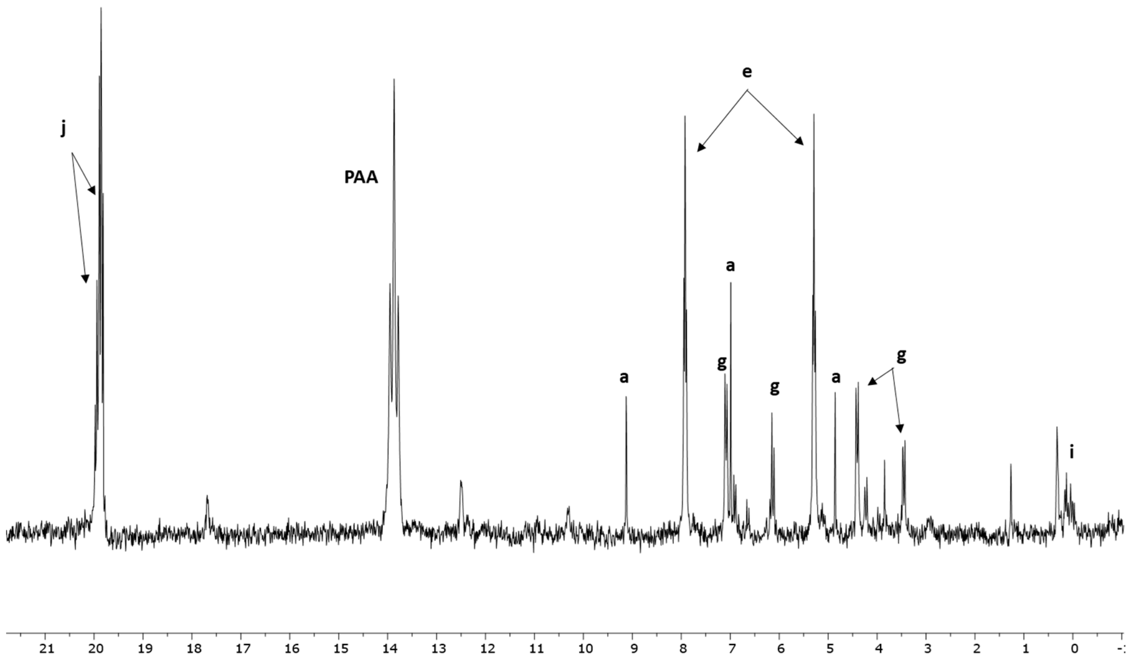

Figure S7. ^{31}P-NMR, H-coupled spectrum of a reaction sample containing adenosine (sample Ad-UAFW-1, Table 1), IPF solution and UAFW. Where peak around 14 ppm is 0.1M phosphonoacetic acid (PAA) that was used as an internal standard.

Quantification by internal standard

The yields of some of the reactions were also quantified by using 0.1M PAA (phosphonoacetic acid) as done previously (for details see ref. 48 in the manuscript and its supporting information). Therefore, this will be discussed here briefly.

The reaction sample was mixed with 0.5 mL of D_2O and 0.5 mL DI water having a total volume of 1 mL. Out of this solution mixture, about 300 μL was added to a clean NMR tube and finally, 9 μL of 0.1M phosphonoacetic acid solution was added to it. The total volume of the solution (mixture) in the NMR tube was 309 μL. The NMR tube was manually shaken to ensure the complete mixing of the solutions. Since the molarity of the internal standard was known (0.1M) the relative molarities of each peak of the organic-P compounds were calculated by employing peak integration method [48]. The relative molarities were based on the dissolved organic-P compounds and their % yields were calculated based on the peak integration method (see reference 51 and its SI).

The yields (%) were based on the amount of phosphorus (P) present in the solution (limiting reactant in this case).

The final no of moles of P in the solution= 0.00004 moles of P (see SI of reference 48).

Sample calculations by using internal standard (Sample Ad-UAFW-1, Table 1)

Molarity of internal standard (PAA) = 0.1 M

Volume of PAA added to NMR tube = 9 μL

No of moles of PAA = Molarity × volume (L) = $0.1 \times 9 \times 10^{-6}$

$= 0.9 \times 10^{-6}$ moles

Volume of solution in the NMR tube = 300 μL or 0.0003 L

Total Volume = Volume of solution in the NMR tube + Volume of 0.1 M (PAA added)

= 300 + 9= 309 μL or 0.000309 L

Molarity of PAA in the NMR tube = No of moles / Total volume (L) = $\frac{0.9 \times 10^{-6}}{0.000309}$

= 0.0174 M

% relative abundance (%) of PAA in the sample = 25%

This establishes 25% = 0.0174 M in 0.000309 L NMR tube solution

(a) Molarity of 2',3'-cyclic AMP in the NMR tube

Relative abundance (%) of 2',3'-cyclic AMP in the sample Ad-UAFW-1 = 23 %

Hence molarity of 2',3'-cyclic UMP in the NMR tube relevant to the PAA= $\frac{0.0174 \times 23}{25}$

= **0.016 M**

(b) Molarity of 2',3'-cyclic UMP in rehydrated solution

Molarity of 2',3'-cyclic UMP in NMR tube = Molarity of 2',3'-cyclic UMP in rehydrated solution

$$\mathbf{M_1V_1=M_2V_2}$$

$\frac{0.016 \times 0.000309}{0.001}$ = 0.0049 M = Molarity in the rehydrated solution (where 0.001 is the rehydrated solution vol.)

No of moles of 2',3'-cyclic UMP = Molarity × volume (L) = 0.0049 × 0.001

= 0.00000494 moles

% yield = No of moles of product/ No of moles of limiting reactant (see **SI of ref. 48**) × 100

$$= \frac{0.00000494}{0.00004*} \times 100 = 12.36\%$$

* where 0.00004 moles relate to the number of moles of p (IPF solution). This number was obtained from the back calculations. The details are given in the reference 48.

% yield of 2',3'-cyclic UMP was therefore around 12.36%

^{31}P-NMR peaks for each C-O-P compounds were related with the 0.1 M PAA, the molarities, and the final yields were individually calculated as described above.
